# Probing and Leveraging the Structural Heterogeneity of Nanomaterials for Enhanced Catalysis

**DOI:** 10.1021/acsnanoscienceau.2c00057

**Published:** 2023-01-27

**Authors:** Rui Yang, Zhenghong Bao, Yifan Sun

**Affiliations:** † Frontiers Science Center for Transformative Molecules, School of Chemistry and Chemical Engineering, Shanghai Jiao Tong University, Shanghai 200240, China; ‡ Biomaterials, Bioengineering & Nanotechnology Laboratory, Department of Orthopaedics, West Virginia University, Morgantown, West Virginia 26506, United States

**Keywords:** structural heterogeneity, nanocatalysis, surface and bulk, local and average structures, catalyst restructuring

## Abstract

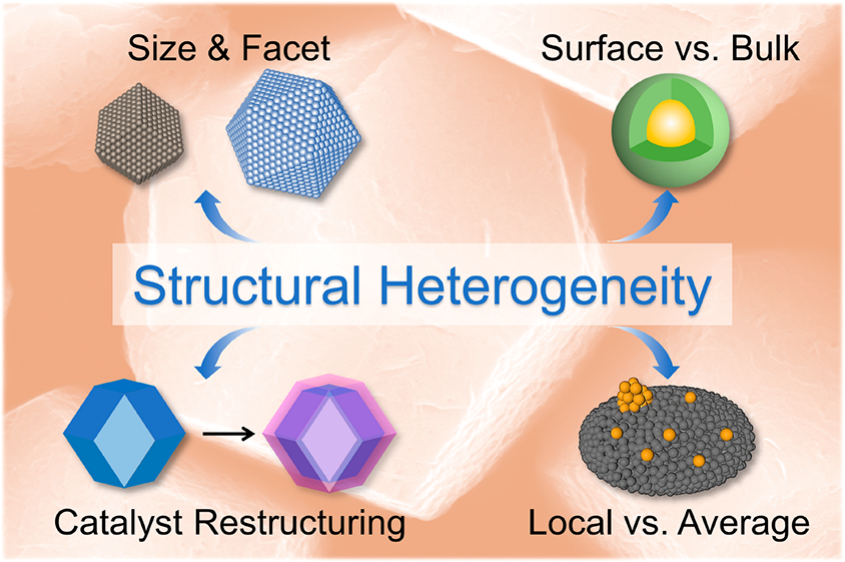

The marriage between nanoscience and heterogeneous catalysis has introduced transformative opportunities for accessing better nanocatalysts. However, the structural heterogeneity of nanoscale solids stemming from distinct atomic configurations makes it challenging to realize atomic-level engineering of nanocatalysts in the way that is attained for homogeneous catalysis. Here, we discuss recent efforts in unveiling and exploiting the structural heterogeneity of nanomaterials for enhanced catalysis. Size and facet control of nanoscale domains produce well-defined nanostructures that facilitate mechanistic studies. Differentiation of surface and bulk characteristics for ceria-based nanocatalysts guides new thoughts toward lattice oxygen activation. Manipulating the compositional and species heterogeneity between local and average structures allows regulation of catalytically active sites via the ensemble effect. Studies on catalyst restructurings further highlight the necessity to assess the reactivity and stability of nanocatalysts under reaction conditions. These advances promote the development of novel nanocatalysts with expanded functionalities and bring atomistic insights into heterogeneous catalysis.

As finite ensembles of atoms, nanomaterials exhibit a multitude of size-dependent properties that are particularly attractive for advanced heterogeneous catalysis. The inherent high surface-to-volume ratio gives rise to high surface areas, with the enriched undercoordinated sites boosting catalytic activities. The pronounced surface effects, benefiting from the ease in constructing various types of chemical bonds at the nanoscale, greatly expand the tunability of selectivity. Stability can also be enhanced through interface engineering, as evidenced for the core–shell or yolk–shell nanocatalysts.^1,2^ The rapid development of nanocatalysis in the past few decades has showcased that the design and optimizing principle of nanocatalysts can be applied to thermal-, electro-, and photocatalysis, surpassing the intrinsic limitation of different driving forces.

Despite the progress, the inherent complexity of nanoscale solids makes it challenging to unambiguously identify the associated structure–property correlations. In homogeneous catalysis, chemical bonds are formed at the same angstrom scale as molecular catalysts. In contrast, the molecular reactants and products in heterogeneous catalysis are in a much smaller dimension than that of the nanocatalysts.^3^ This dimension mismatch between the molecular species and the nanocatalysts, together with the size-dependent geometric and electronic characteristics of nanosolids, brings multilevel deviations between the ideal atomically ordered model system and the real nanomaterial samples. These variations give rise to inconsistent and sometimes even contradictory conclusions regarding the seemingly same materials. The corresponding uncertainties make it challenging to extend the obtained insights to a wider scope of catalytic materials, or establish universal principles for catalyst design. Substantial efforts, including controlled synthesis, atomically resolved characterization approaches, and high-throughput computational screening, have been directed toward building well-defined nanostructures with minimized batch-to-batch variability. However, reaching the molecular details that homogeneous catalysis has been designed and interpreted is likely an impossible mission for heterogeneous nanocatalysts, at least in the short term. It thus becomes crucial to acknowledge, understand and try to leverage the inescapable structural heterogeneity in nanomaterials to resolve the encountered issues in catalytic reactions.

The historic development of strong metal–support interaction (SMSI) is an intriguing example benefiting from diving into the structural heterogeneities of nanocatalysts. Back in 1978, Tauster et al. found that the chemisorption properties of probe molecules like H_2_ and CO on the TiO_2_-supported platinum group metal (PGM) nanoparticles vanish upon high-temperature reduction treatments, which was initially ascribed to the reduction-induced formation of metal alloys or metal hydrides.^4^ The development of knowledge and techniques in surface chemistry later shed light on the surface inhomogeneity of the supported PGM nanoparticles, as the reduction treatment results in the formation of the Ti^3+^-containing suboxide overlayers that block the adsorption active sites.^5^ The constructed surface-confined oxide overlayer/metal interfaces are distinct from the individual metal and metal oxide constituents, and fundamentally determine the geometric and electronic characteristics of the supported nanocatalysts. For a long time, SMSI was considered to increase the stability yet lower the activity due to the encapsulation architecture. Studies in recent years have indicated that the encapsulating overlayer, when being reduced to atomically thin, can also be inhomogeneous.^6,7^ Local defects and pore engineering allow reactant molecules to reach the metal surfaces, triggering catalytic conversions. This also opens doors for selectivity tuning through modulating the interfacial geometric (nanoconfinement) and electronic (charge transfer) aspects.^8^ This series of discoveries manifest the advances empowered by unfolding the structural heterogeneities involving the surface and interface effects, as well as local lattice imperfections.

Herein, we look into the origin, classification, and features of structural heterogeneities in nanomaterials (Figure 1), which can be probed and leveraged to promote enhanced catalysis. We start from the analyses of size and facet of nanocrystals as well as the associated impacts on the surface adsorption and catalytic properties. Differentiation of surface and bulk characteristics is further demonstrated using nanoceria-based catalysts as examples, where the spatial distribution of aliovalent metal substituents and oxygen defects are key to sparking efficient lattice oxygen activation. We then move to the careful comparison of local and average structures, showing that appraising and harnessing the compositional and species uniformity via the ensemble effect is useful for accommodating a wider scope of catalytic reactions. Restructuring of nanocatalysts under reaction conditions is also illustrated as an unconventional route toward building active and robust catalytic sites. In the end, we provide our own perspective in combining synthesis, characterization, and measurement efforts to explore fundamentally distinct and conceptually novel systems, such as chiral nanoparticles and high-entropy materials (HEMs). The capability to unveil and decouple entangled factors causing structural heterogeneities in nanocatalysts lays the foundation for accessing better catalysts with improved activities, selectivities, and stabilities.

**Figure 1:**
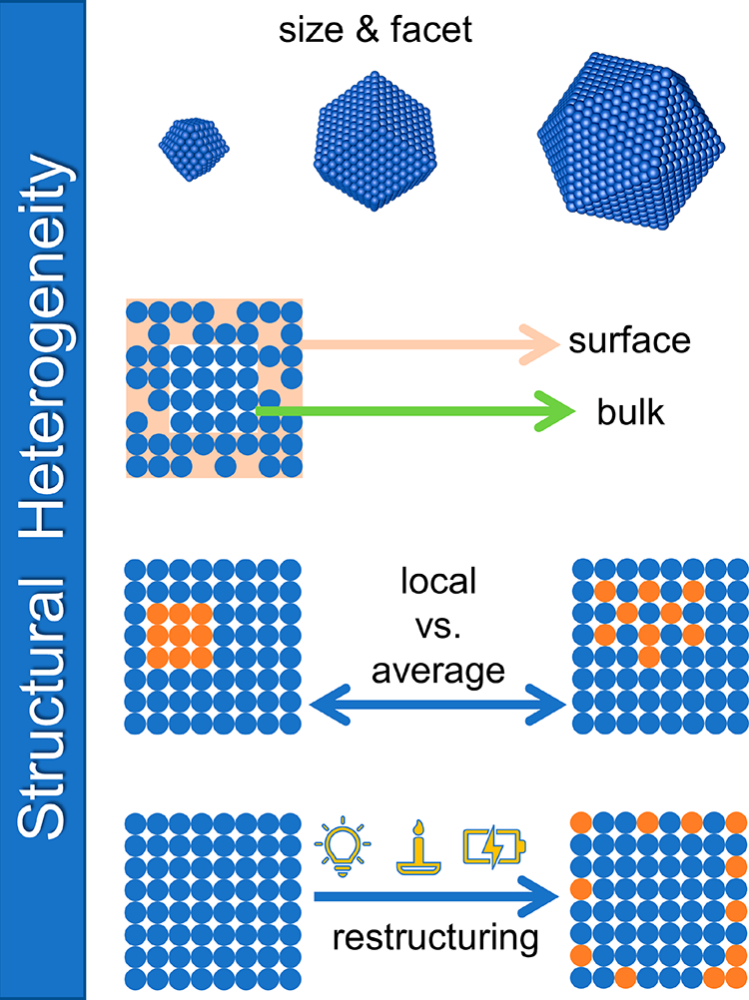
Schematic summarizing the four types of structural heterogeneity in nanocatalysts: difference in size and facet, surface and bulk characteristics, local vs average structures, and catalyst restructuring.

## The Influence of Size and Facet

The fundamental difference between nanostructured and bulk materials is size. Back in 1965, Hardeveld et al. found that the nitrogen adsorption results of Ni, Pt and Pd are strongly dependent on the crystallite size.^9^ This inspires enormous studies that play with the combination and permutation of catalysts and reactions to uncover the size effect for catalysis. Surface area used to be the touchstone of catalytic activities. It is clear now that the higher concentration of the surface-exposed coordination unsaturated sites that are favorable for molecular adsorptions pull the trigger. It is thus pivotal to quantify the number and identify the type of the catalytically active sites, serving as a prerequisite to elaborate the influence of size on catalytic performances.

The definition of size varies for different nanocatalyst systems, and smaller sizes do not necessarily guarantee larger active surface area or more active sites. For instance, the supported nanoparticles may sink or embed into the underlying substrates upon thermal treatments,^10^ making it difficult to estimate the active area by solely comparing the size of pristine particles. In addition, there is a basic difference between domain and particle sizes. The grain boundaries with one-dimensional line defects in polycrystalline materials bring extra active sites that can be overlooked, as evidenced in the case of grain-boundary-rich Au and Cu nanocrystals for electrochemical conversion of CO_2_.^11,12^ It is thereby formidably challenging to determine the number of the active sites. Techniques that rely on the adsorption of molecular probes, such as chemisorption and diffuse reflectance infrared Fourier transform spectroscopy (DRIFTS) in thermocatalysis, as well as double-layer capacitance (*C*_dl_) measurement and hydrogen underpotential deposition (HUPD) in electrocatalysis, bring more accurate and reliable results for catalytic studies.^13−15^

**Figure 2:**
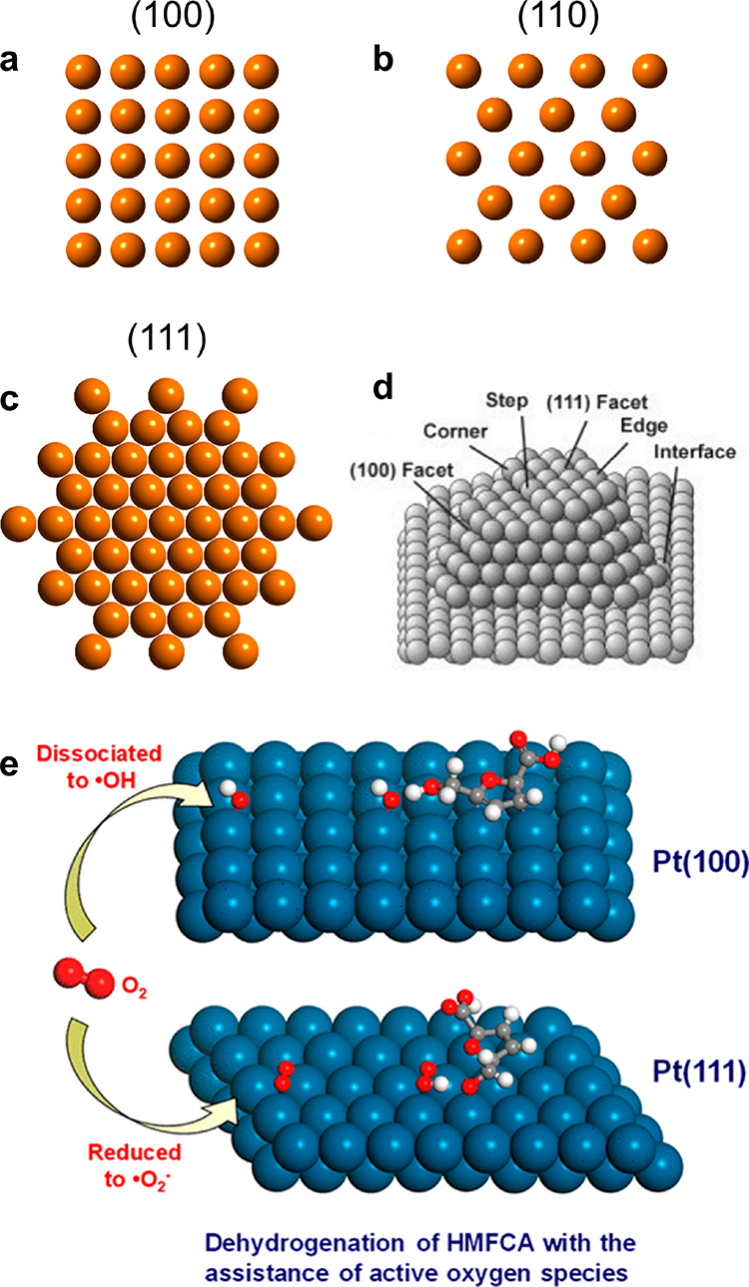
Difference in the exposed facet of nanocatalysts. Atomic models showing the surface arrangements of the (a) (100), (b) (110), and (c) (111) facet of the fcc metals. (d) Schematic highlighting the coordinatively unsaturated sites. Reprinted from ref (16). Copyright 2004 American Association for the Advancement of Science. (e) Schematics showing the preferential formation of ·OH and ·O^2–^ species via oxygen activation on the Pt (100) and (111) facet, respectively. The ·OH generated on the Pt (100) facet brings higher activities catalyzing HMF oxidation. Reprinted from ref (17). Copyright 2019 American Chemical Society.

In addition to size, the exposed facet imposes a substantial impact on the number and type of the catalytically active sites. The binding mode of the molecular species is underpinned by the exposed atomic arrangements (Figure 2a-c), and multiple adsorbing species may coexist. Even on the same facet, several types of the coordinately unsaturated sites, such as, corner, step, edge, terrace, etc., lead to distinct binding sites for molecular species (Figure 2d).^16^ For instance, during the oxidation of 5-hydroxymethyl-2-furfural (HMF) on the Pt nanocrystals, molecular O_2_ is inclined to form ·OH and ·O^2–^ on the Pt (100) and (111) facets, respectively.^17^ Compared with ·O^2–^, the ·OH species formed on the Pt (100) facet exhibits stronger oxygen activation capabilities, catalyzing aerobic oxidation of HMF via the dehydrogenation pathway (Figure 2e). For ceria (CeO_2_), the oxygen vacancy formation energy of the commonly observed (111), (110), and (100) facet is computationally estimated to be 2.60, 1.99, and 2.27 eV, respectively, suggesting facet-dependent generation and stabilization of oxygen vacancies.^18^ The adsorption, activation, and conversion of molecular species varies for the nanocatalysts with different sizes or exposed facets, ultimately regulating the catalytic reactivities.

## Differentiating Surface and Bulk Characteristics

Besides size, another layer of structural heterogeneity of nanocatalysts originates from surface and bulk characteristics. Surface atoms are generally coordinatively unsaturated with respect to the equilibrium bulk structure.^19,20^ The surface effects are further pronounced for nanomaterials, as surface and subsurface atoms are inclined to undergo reconfiguration owing to the increased surface free energy.^21^ Catalysis has long been believed to be governed by surface chemistry, while more and more evidence has suggested that the bulk counterparts also play a nonnegligible role in determining the catalytic activities,^22^ particularly for nanostructured metal oxides. In addition to surface oxygen species, activation and transport of oxygen deep into the bulk lattice afford additional active oxygen species contributing to molecular conversions. For instance, the bulk lattice oxygen in metal oxides becomes available at sufficiently high temperatures, migrating to surfaces and boosting the methane oxidation rates via the intrafacial oxygen mechanism.^23^

**Figure 3:**
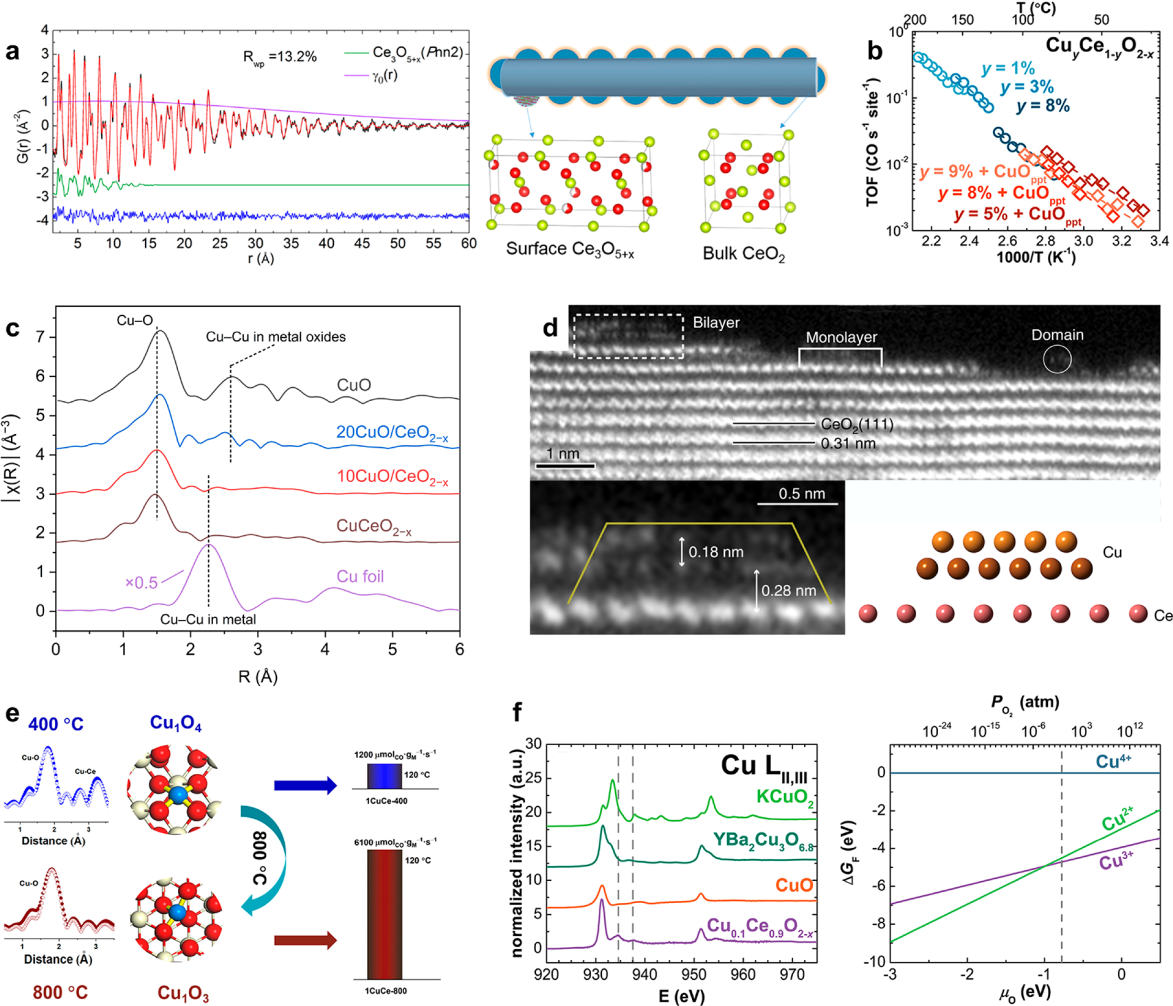
Surface and bulk characteristics of ceria-based nanocatalysts. (a) Neutron scattering data with PDF analysis for the CeO_2_ nanorods, where Ce_3_O_5+x_ is identified as the surface phase plus the bulk fluorite structure of CeO_2_. Reprinted from ref (30). Copyright 2021 American Chemical Society. (b) Comparison of turnover frequencies (TOFs) of CO oxidation catalyzed by the Cu_*y*_Ce_1–*y*_O_2–*x*_ samples. Segregation of the secondary CuO phase has negligible impact on the reaction kinetics, revealing that the Cu-substituted Cu_*y*_Ce_1–y_O_2–x_ part is the true active site while the phase-segregated CuO serves as spectators. Reprinted from ref (39). Copyright 2016 American Chemical Society. (c) Fourier transforms of the extended X-ray absorption fine-structure spectroscopy (FT-EXAFS) profiles at the Cu K-edge for the interface-engineered CuO/CeO_2–*x*_ and substituted CuCeO_2–x_ samples, as well as Cu and CuO references, showing the copper atoms are ultimately stabilized on the CeO_2_ surface regardless of the synthesis pathway. Reprinted with permission under a Creative Commons CC BY License from ref (38). Copyright 2022 John Wiley and Sons. (d) High-angle annular dark-field scanning transmission electron microscopy (HAADF-STEM) images and atomic models showing the bilayer copper cluster interfaced with the underlying CeO_2_ support. Reprinted with permission under a Creative Commons CC BY-NC License from ref (40). Copyright 2019 Nature Publishing Group. (e) Scheme showing the CO oxidation activity boost from the Cu_1_O_4_ geometry prepared at 400 °C to the coordination-unsaturated Cu_1_O_3_ structure obtained through calcination in air at 800 °C, which manifests the feasibility to alter the structural heterogeneity with temperature control. Reprinted from ref (41). Copyright 2019 American Chemical Society. (f) Oxygen K-edge and copper L-edge X-ray absorption spectroscopy (XAS) data of the Cu_0.1_Ce_0.9_O_2–*x*_ sample and the CuO, YBa_2_Cu_3_O_7−δ_, and KCuO_2_ references (left). The resemblance of the charge-transfer multiplet satellite peaks demonstrates the existence of Cu^3+^ species in the copper-substituted ceria nanocatalysts. Calculated phase diagram with the Gibbs formation energy (Δ*G*_F_) as a function of oxygen chemical potential for the Cu^2+^, Cu^3+^, and Cu^4+^ species in the Cu^(4–*x*)+^_2_Ce_34_O_72–*x*_ structure (right). Reprinted from ref (45). Copyright 2014 American Chemical Society.

We here take CeO_2_-based nanocatalysts as an example. As one of the most widely applied catalyst materials, CeO_2_-based catalysts exhibit tunable reactivities underpinned by the Ce^4+^/Ce^3+^ redox pair along with oxygen defect formations.^24,25^ For pristine CeO_2_, various types of oxygen defects tend to enrich on the surface, as coordinatively unsaturated sites are energetically favorable when being exposed to the environment compared with those being confined within the bulk lattice.^26^ This also facilitates oxygen release/uptake into/from the atmosphere for oxidation reactions. Development of cutting-edge characterization techniques provides direct evidence highlighting the surface/bulk heterogeneities in pristine CeO_2_.^27,28^ Combining aberration-corrected scanning transmission electron microscopy (STEM) with electron energy loss spectroscopy (EELS) techniques, Ikuhara et al. applied the Ce^3+^/Ce^4+^ (Vo) distribution as the descriptor for evaluating the concentration of oxygen defects on the surface and in bulk of the (100)-terminated, single-crystalline CeO_2_ nanocubes.^29^ An obvious EELS peak shift to lower energies was only observed in the several outmost atomic layers of the 9.7 nm CeO_2_ nanocrystals, whereas the similar peak shifts were found across the whole 5.4 nm CeO_2_ nanocrystals. Quantitative analyses of the Ce^3+^-like species further showcase the size-dependent geometric and electronic heterogeneities of oxygen defects. Complementary to the microscopic imaging data, neutron scattering coupled with pair distribution function (PDF) analyses permits a more precise structure interpretation of the surface and bulk oxygen defects (Figure 3a).^30^ Two different types of oxygen defects were precisely identified, the partially reduced Ce_3_O_5+x_ Schottky-type defects dominating on the surface, and the interstitial Frenkel-type oxygen vacancies existing in bulk. Solid-state nuclear magnetic resonance (ssNMR) also allows quantitative identification of the chemical state of surface Ce species, enabling judicious selection of the exposed CeO_2_ facet catalyzing acid–base reactions.^31^

The situation is complicated by incorporating aliovalent elements into ceria. The rationale is to introduce transition metal elements such as Cu, Ni, and Co carrying lower charges than that of Ce^4+^, to promote oxygen defect formation through charge neutralization and lattice distortion.^32^ The incorporated transition metal species also contribute active centers that trigger thermal catalysis, electrocatalysis and photocatalysis.^33,34^ However, the local structures and distributions of the introduced aliovalent atoms have remained elusive. Taking the copper-ceria nanocatalysts as an example, homogeneous Cu–Ce–O_*x*_ solid solutions,^35^ CeO_2_-suppored Cu (CuO_*x*_) clusters with Cu–Ce–O interfaces,^36^ or a mixture of both^37^ have all been reported. These instances reflect different levels of surface/bulk heterogeneities, which inspire efforts to explicate the copper–ceria interactions and their impact on lattice oxygen activation for catalytic reactions.

Due to the structural complexity of mixed metal oxides and composition-dependent phase stabilities, the boundary between lattice substitution and interface engineering is blurred in the copper–ceria system. Thermal annealing of the physically mixed, presynthesized Cu and CeO_2_ nanocrystals yields atomically dispersed Cu species anchored on CeO_2_, which resembles the atomic structure of the Cu-substituted CeO_2_ nanocrystals prepared via the one-pot synthesis (Figure 3c).^38^ The strong interactions between Cu and the underlying CeO_2_ support prompts interface-mediated decomposition, migration, and redispersion of Cu via a solid–solid route. This implies that regardless of the synthesis pathway, either aliovalent doping or interface construction, surface substitution of Cu on CeO_2_ would be the ultimate, energetically favorable state with low Cu contents (∼ ≤10 mol %). Further elevating the copper amount induces the CuO phase segregation. For CO oxidation, these phase-segregated CuO precipitates function as spectators while the true active sites remain to be the surface-substituted Cu_*y*_Ce_1–y_O_2–x_ phase (Figure 3b).^39^ In a recent study, Shen and co-workers uncovered the two-dimensional perimeter structure of the catalytically active copper–ceria interface (Figure 3d).^40^ The top and bottom layer is identified as Cu^0^ atoms and copper clusters in the form of Cu^+^–O_v_–Ce^3+^, respectively, highlighting the atomic-level heterogeneity even for the same elemental species (Cu) originating from the difference in the interfacial bonds. The Cu^+^ site enables CO adsorption, while the neighboring O_v_–Ce^3+^ site facilitates H_2_O dissociation, giving rise to enhanced low-temperature water–gas shift reaction (WGSR) capabilities via a site cooperation mechanism. These studies suggest that the description of Cu–O–Ce solid solutions or interfacial CuO_*x*_/CeO_2–*x*_ heterostructures may not comprehensively describe the local structures of the copper–ceria nanocatalysts. Deposition or substitution of Cu species on the surface and subsurface lattice of CeO_2_ can be a more accurate description that highlights the surface/bulk heterogeneities. This is important for mechanistic studies constructing surface model structures with relaxed slabs or periodic bulk structures with repeating unit cells.

A following question is how to modulate the structural heterogeneity of the copper–ceria catalysts to accommodate a wider scope of reactions. Two routes playing with the thermodynamic driving forces emerge. The first is to alter the synthesis or processing temperature. Common treatment temperatures are generally around or below 500 °C, with the goal to realize controlled phase transformation, remove the protective ligands and/or enhance the metal–support interactions. Higher processing temperatures, i.e., ≥800 °C, induce distinct local structures with variable phase stabilities for the copper–ceria system. For example, calcinating copper–ceria nanocatalysts in air at 800 °C transformed the as-formed Cu_1_O_4_ geometry to the coordination-unsaturated Cu_1_O_3_ structure, exhibiting compelling catalytic activity and stability for CO oxidation (Figure 3e).^41^ With higher Cu contents, the 800 °C annealing in air brings unique interfacial sites between the supported subnanometer CuO_*x*_ clusters and the Cu-doped ceria thin layer.^37^ In addition to temperature control at the equilibrium state, mechanochemistry trigger chemical reactions with ultrahigh local energies at room temperature, which also opens the door to manipulate the Cu dispersion on the surface and in bulk of CeO_2_.^42^ The second strategy is compositional engineering, as the synergistic interactions among multiple components may alter the entropy–enthalpy correlations. Simultaneous incorporation of copper and iron in ceria provides additional active sites for lattice oxygen activation and release, substantially elevating the WGSR activity and stability.^43^ The emergence of high-entropy oxides (HEOs) containing five or more cations confined in a single lattice provides a versatile playground to exploit the high-entropy effect. Simultaneous incorporation of Cu, Co, Fe, Ni, and Mn in ceria nanocrystals modulates the local structural heterogeneity, inducing the formation of surface-confined atomic HEO layers.^44^ The enhanced covalency of the transition-metal–oxygen bonds at the HEO–CeO_2_ interface promotes surface oxygen vacancy formation, leading to efficient lattice oxygen activation and replenishment catalyzing CO oxidation reactions.

The above-mentioned surface/interface effects also pave the road toward formation and stabilization of exotic chemical species that are appealing for catalytic reactions. For example, Shao-Horn and colleagues reported the presence of Cu^3+^ species in the Cu- substituted CeO_2_ nanocrystals (Figure 3f), which substantially lowers the formation energy of oxygen vacancies.^45^ This is surprising, as Cu^3+^ existing in cuprate materials such as YBa_2_Cu_3_O_7−δ_ and KCuO_2_ are typically air sensitive and have a strong inclination toward conversion to Cu^2+^.^46^ The Cu^3+^ in Cu_*y*_Ce_1–*y*_O_2–*x*_ not only is stable but also participates in CO oxidation reactions, facilitating the formation and refilling of oxygen vacancies via the Mars–van Krevelen mechanism. While the origin and electronic structures of the odd Cu^3+^ species in the copper-ceria nanocatalysts remain in debate,^47^ structural heterogeneity with pronounced surface effects of the ceria lattice is a pivotal aspect to be considered in future studies.

Besides ceria-based nanomaterials, a wider array of nanomaterials exhibit surface- and bulk-dependent catalytic behaviors that intrigue both experimental and computational studies. Oxide perovskites exhibit tunable redox properties, oxygen mobilities, and ionic conductivities that are appealing for catalysis.^48^ Using synchrotron-based in situ X-ray diffraction (XRD), Penner and co-workers probed the bulk phase transformation of LaNiO_3_ in the atmosphere of dry methane reforming (DMR).^22^ The results inarguably prove the dynamic structural change from LaNiO_3_ to oxygen-deficient LaNiO_2.7_ and LaNiO_2.5_, transient formation of La_2_NiO_4_ with exsoluted La_2_O_3_ and Ni species, and final stabilization with the Ni/La_2_O_3_/La_2_O_2_CO_3_ heterostructures, all of which are correlated to the corresponding DMR activities. This manifests the feasibility of turning to the bulk crystalline phase to unfold the surface structure–performance correlations. Similar discussions were provided for the single/double perovskites, where regulation of surface and bulk properties gives rise to optimized oxygen evolution reaction (OER) performances.^49^ Computational studies sampling the outmost surface structures and inner bulk lattices reveal more detailed differences regarding chemical ordering, lattice strain, density of states, as well as thermodynamic-driven surface segregation and bulk phase transition for binary metal nanoalloys and metal carbide nanoparticles.^21,50^

## Local vs Average Structures

**Figure 4:**
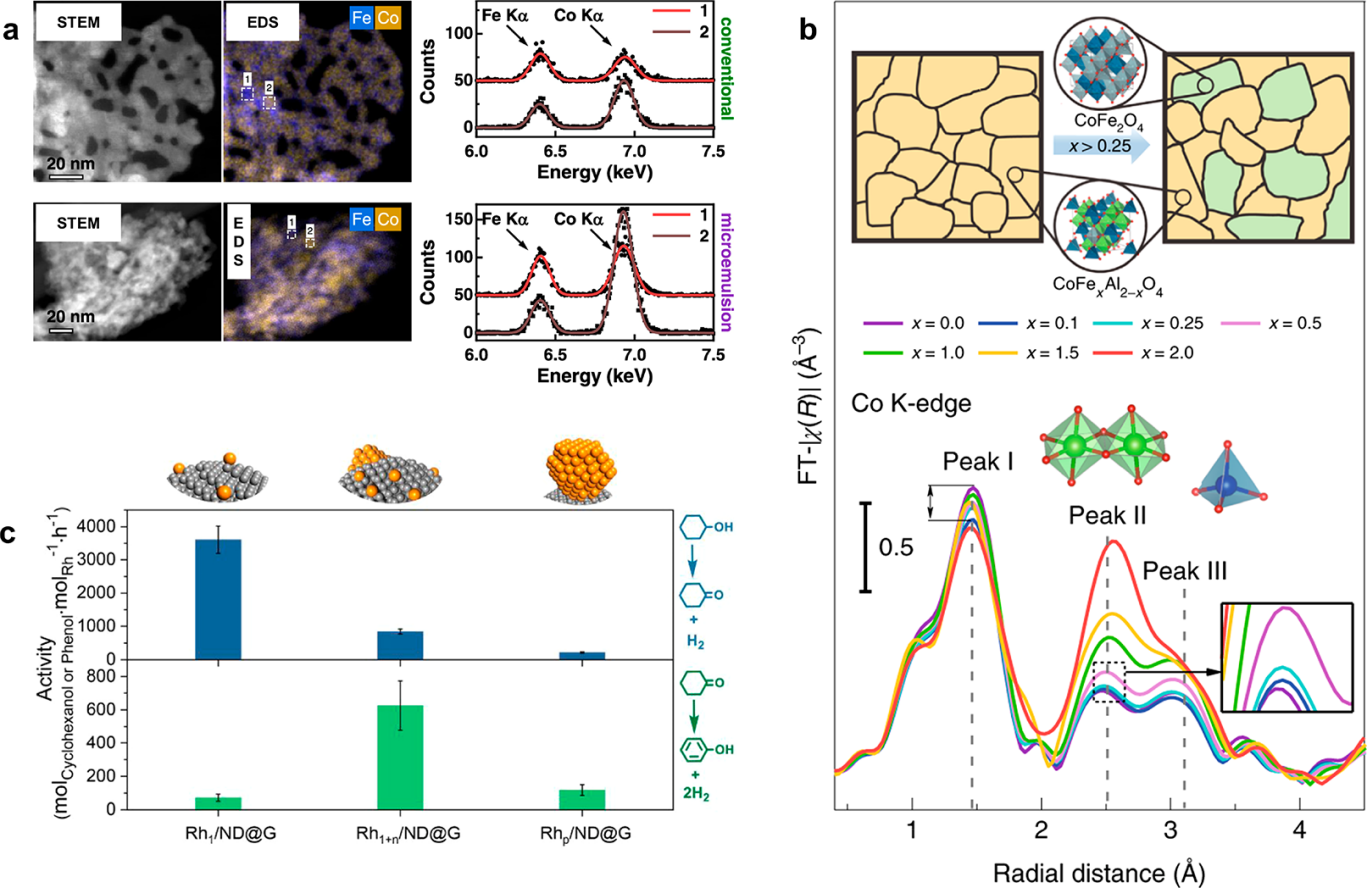
Local vs average structures involving compositional and species heterogeneities. (a) ADF-STEM image, energy dispersive spectroscopy (EDS) element maps and EDS spectra extracted from the highlighted regions for the conventional and microemulsion Co_2_FeO_4_ samples, indicating a nonuniform distribution of Co in the microemulsion sample. Adapted from ref (51). Copyright 2022 American Chemical Society. (b) FT-EXAFS data of the Co K-edge of CoFe_*x*_Al_2–*x*_O_4_ sample showing the CoFe_2_O_4_ phase segregation with higher Fe contents (*x* > 0.5). Reprinted with permission under a Creative Commons CC BY-NC License from ref (52). Copyright 2019 Nature Publishing Group. (c) Metal-normalized activity of the cyclohexanol-to-cyclohexanone (above) and cyclohexanone-to-phenol (below) conversion catalyzed by the Rh single-atom (Rh_1_/ND@G), nanocluster (Rh_1+n_/ND@G), and nanoparticle Rh_p_/ND@G catalyst, suggesting observable activity difference in catalyzing the two steps for the cyclohexanol dehydrogenation reaction. Reprinted from ref (54). Copyright 2022 American Chemical Society.

Catalyst preparation is expected to yield uniform nanostructures in the homogeneous reaction medium. However, the solution- or gas-phase microenvironment dynamically evolves at different synthesis stages, ultimately producing several types of kinetically hindered nanostructures with similar thermodynamic formation barriers. It is thereby crucial to probe the associated compositional and species heterogeneities dictated by the ensemble effect. Within the same batch of nanocatalysts, some domains directly contribute to efficient molecular conversions, while the others exhibit lower activities or even function as inactive spectators. It has remained challenging to pinpoint the correlation between the local structures and average catalytic performances. Taking cobalt ferrites (CoFe_2_O_4_) with the inverse spinel structure as an example, equal amounts of the Co^2+^ and Fe^3+^ cations occupy the octahedral sites whereas the rest Co^2+^ cations reside in the tetrahedral sites. As the spinel and inverse spinel structures share the same composition and similar structures, it is common for the Fe^3+^ cations to migrate to the neighboring tetrahedral site to substitute Co^2+^. This adds to the disorder within the oxide lattice and facilitates oxygen activation for efficiently catalyzing OER. Cuenya and colleagues prepared two structurally equal Co_2_FeO_4_ spinels with nominally identical stoichiometry using the conventional coprecipitation and microemulsion-assisted coprecipitation method, respectively.^51^ Interestingly, the microemulsion Co_2_FeO_4_ exhibits intrinsically higher activities and faster OER kinetics relative to those of the other sample, which is attributed to the pronounced Co-enrichment in the nanoscale domains glued by the secondary Co-containing amorphous phase (Figure 4a). These local structural differences can hardly be observed using bulk techniques like XRD and inductively coupled plasma optical emission spectrometry (ICP-OES) that extract average structural information. Similar local heterostructures comprising CoFe_2_O_4_ and CoFe_*x*_Al_2–x_O_4_ domains were observed when introducing Fe into aluminum cobalt oxide (CoAl_2_O_4_) (Figure 4b).^52^ Fe substitution in CoAl_2_O_4_ alters the coordination environment of the Co site (octahedral vs tetrahedral). The modified local atomic structures with decreased Co–O coordination number and lower formation barrier for oxygen vacancies collectively promote the OER process. Note that the interfaced nanoscale heterostructures constructed via cation substitution facilitate surface reconstruction and creation of the oxyhydroxide active sites, which will be discussed in a later section.

For the as-prepared nanocatalyst batches, coexistence of the metal single atoms, atomic clusters and nanoparticles possessing different sizes, facets and accordingly coordination environments, is almost inevitable. This is mainly caused by the higher mobilities and reactivities of nanoscale and atomic-level species relative to those of the bulk counterparts. The species heterogeneity tends to exploit the potential of one particular species for triggering simple reactions with a single rate-determining step (RDS).^53^ Therefore, how to increase the utilization rate of the distinct active components in the nanocatalysts with inherent species heterogeneity is important yet remains challenging. Recent work from Ma and co-workers resolved this issue in a diametrically opposite way, simultaneously exploiting the single atoms and ensemble sites (cluster and nanoparticle) catalyzing multistep reactions.^54^ The activity of cyclohexanol dehydrogenation was optimized by combining the isolated Rh atoms (Rh_1_) that efficiently transform cyclohexanol to cyclohexanone and the Rh ensemble sites for the successive reaction to yield phenol (Figure 4c). It is worth mentioning that the Rh_1_ sites are almost inactive for the second step, highlighting the indispensability of the Rh ensemble sites in driving the stepwise two-step cyclohexanol dehydrogenation to phenol. Judicious selection of tandem and cascade reactions directs a new approach to exploit the species heterogeneity, which is a synthesis hindrance for both lab- and large-scale manufacturing, for catalytic utilities.

## Catalyst Restructuring

The above discussion is based on the structural heterogeneities of the as-synthesized nanocatalysts. Meanwhile, nanocatalysts are inclined to undergo restructuring under external stimuli during catalytic reactions, triggered by the nonequilibrium parameters such as reaction temperature, atmosphere, and/or surface adsorbates. This is different from the cases that involve catalyst pretreatments, such as steam pretreatment of palladium nanoparticles supported on alumina inducing grain boundaries for efficient methane oxidation,^55^ or oxide-derived nanocrystalline Cu boosting the CO electroreduction performance to liquid fuels.^56^ The origin for the structural evolution during catalytic reactions is the dynamic balance between catalytic reactivity and chemical stability, with the driving force of minimizing the surface energy under reaction conditions. The restructuring process alters the heterogeneities in size and facets, surface and bulk, as well as local and average structures of the nanocatalysts, giving rise to modified catalytic performances.

**Figure 5:**
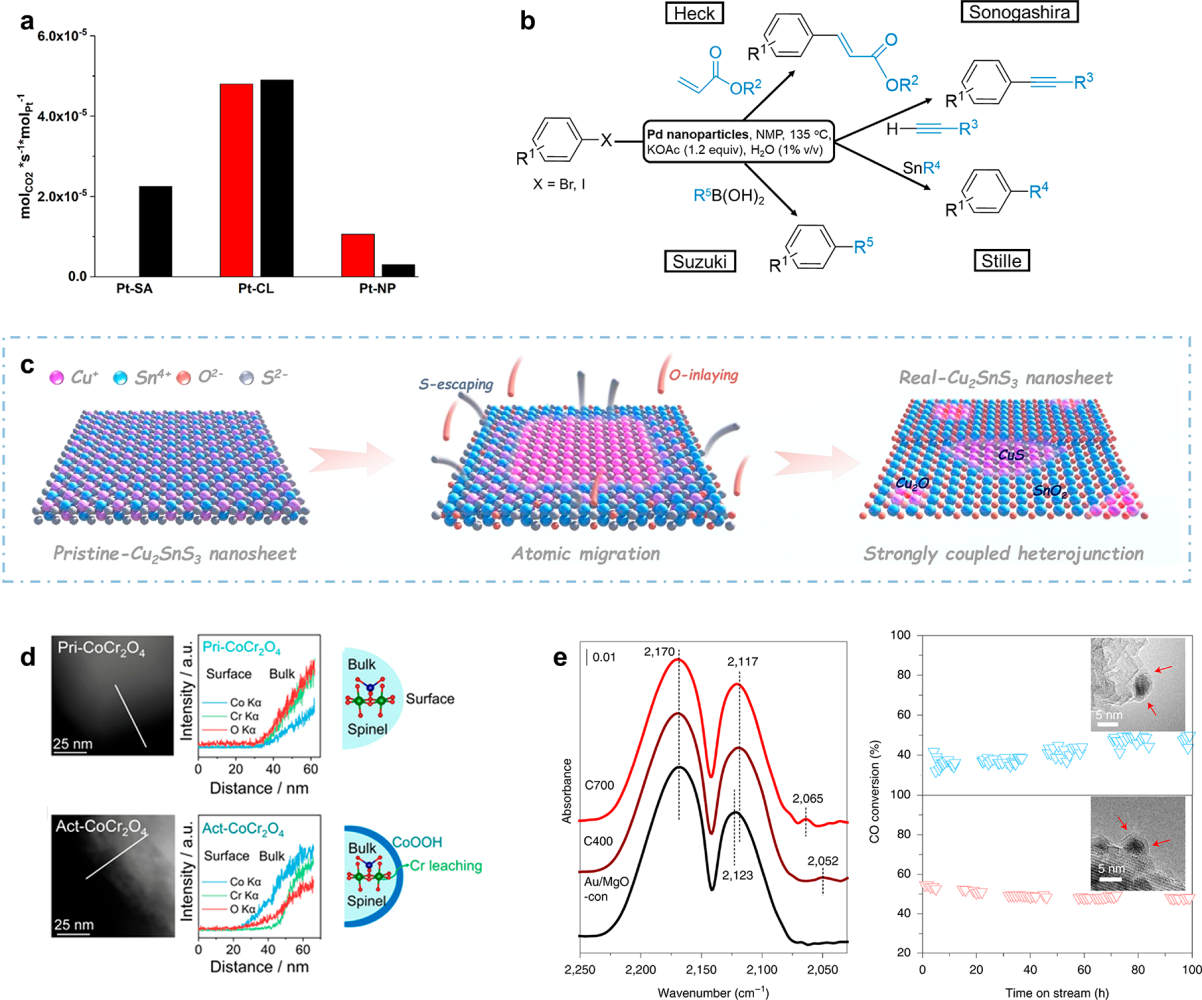
Catalyst restructuring under reaction conditions. (a) Catalytic activity of the Pt single atoms (Pt-SA), clusters (Pt-CL) and nanoparticles (Pt-NP) for CO-assisted NO reduction at 200 K. The red and black bar denotes the CO_2_ production rate right on stream and after 700 s of reaction, respectively. The sharp contrast in activity suggests the transformation of Pt single atoms to clusters that exhibit the highest catalytic activity. Reprinted from ref (57). Copyright 2019 American Chemical Society. (b) Scheme showing the occurrence of the Heck, Sonogashira, Stille, and Suzuki cross-coupling reactions catalyzed by the nanoparticle-transformed Pd clusters in the presence of water. Adapted with permission from ref (58). Copyright 2013 John Wiley and Sons. (c) Schematic showing the in situ transformation of homogeneous Cu_2_SnS_3_ to CuS@SnO_2_ heterostructures interfaced with Cu_2_O under electrochemical CO_2_ reduction conditions. Reprinted with permission from ref (61). Copyright 2021 John Wiley and Sons. (d) STEM-EDS line scan results capturing the distribution of the Co, Cr and O from surface to the bulk counterpart of the pristine (Pri-CoCr_2_O_4_) and activated CoCr_2_O_4_ (act-CoCr_2_O_4_). This reveals the surface Cr leaching accompanied by the surface-confined formation of Co oxyhydroxides (CoOOH), which drastically enhances the OER activity. Reprinted with permission from ref (62). Copyright 2021 John Wiley and Sons. (e) In situ CO DRIFTS data showing the linear adsorption of CO on the Au surface sites (left). CO oxidation activity at 300 °C for the 700 °C-treated Au/MgO nanocatalysts in the dry (top right) and humid (bottom right) atmosphere. The transmission electron microscopy (TEM) images in the insets demonstrates good stability of the atomically thin MgCO_3_ overlayer under reaction conditions. Reprinted with permission under a Creative Commons CC BY-NC License from ref (64). Copyright 2021 Nature Publishing Group.

The change in size or facet of the nanocatalysts during catalytic reactions has been widely encountered. Extensive ex situ and in situ studies have been carried out to unveil the transformation between single atoms and subnanometric clusters and larger particles under reaction conditions. The catalyst–reactant and metal–support interactions are the two main thrusts that alter the relative stabilities of the dispersed or agglomerated species with different sizes, presenting the real working sites. Taking PGM nanocatalysts as an example, different reaction environments give rise to distinct evolution pathways. Supported Pt SACs are prone to agglomerate to Pt nanoclusters and particles that catalyze NO reduction reaction (2NO + 2CO = N_2_ + 2CO_2_) at low temperatures (140–200 K) (Figure 5a).^57^ Only subnanometric Pt clusters allow NO dissociation and CO oxidation simultaneously occurred on the catalyst surface, while larger Pt nanoparticles become poisoned by CO. Catalyzing C–C cross-coupling reactions, ligand-free Pd nanoparticles remain inactive until being leached and converted to the three- or four-atom Pd clusters, which can be stabilized with water molecules (Figure 5b).^58^ Supported Pd nanoparticles also undergo size and facet evolutions during NO/CO deNO_*x*_ cyclings, as evidenced by a reversible sintering and nonoxidative redispersion phenomena.^59^ The intermediate NCO species that adsorb on the Pd surfaces modify the surface stability under reaction conditions, leaving the most stable facet being exposed for catalytic reactions. These structural transformations in size and/or shape under reaction conditions create or enrich catalytically active sites that are generally difficult to access during the synthesis step.

Besides geometric influences, the restructuring process also leads to compositional changes that bring about surface/bulk and local/average heterogeneities. One typical example belongs to the numerous reports of nanostructured metal chalcogenides, nitrides, and phosphides catalyzing OER. It is likely the surface of these nanocatalysts is oxidized to metastable or amorphous metal oxides and/or (oxy)hydroxides under oxidizing potentials, affording the real OER active sites that are no longer chalcogenides, nitrides, or phosphides.^60^ Note that the restructured surfaces of nanocatalysts possess increased structural and compositional complexities, and the core–shell interface needs to be carefully examined to evaluate the electronic effects. Similar surface-confined restructurings are also observed for electrochemical CO_2_ reductions, where the solution environment induces self-adapted phase separation of the Cu_2_SnS_3_ nanocatalyst and produces SnO_2_@CuS and SnO_2_@Cu_2_O heterojunctions (Figure 5c).^61^ Fine-tuning the reaction parameters allows controllable surface restructurings that aid the creation and enrichment of active sites. For example, controllable anodic leaching of Cr in the CoCr_2_O_4_ nanocatalysts exposes the active Co oxyhydroxides accompanied by the formation of oxygen defects, collectively leading to desired OER properties (Figure 5d).^62^ In contrast, pristine CoCr_2_O_4_ is OER inactive, highlighting the power of catalytic restructuring in mastering surface reactivities.

A special case for the reaction-induced structural heterogeneity is adsorbate-induced strong metal–support interaction (A-SMSI). The metal–support interactions can be adjusted by adsorbing certain chemical species during reactions, and the associated electronic charge transfer or geometric encapsulation dramatically modifies the reaction pathways. Christopher and co-workers pioneered the field by discovering the encapsulation of oxide-supported Rh nanoparticles with formate and carbonate-like (HCO_*x*_) permeable adsorbates enables full-range tuning of the CO_2_ hydrogenation products.^63^ Moving beyond reducible oxide supports, which have been regarded as a prerequisite for SMSI, it is now viable to grow oxide overlayers on the MgO-supported Au nanoparticles guided by the reversible reaction of MgO + CO_2_ ⇄ MgCO_3_ (Figure 5e).^64^ This further broadens the applicability scope of SMSI, and the added heterogeneity at the metal/support interface reinforces the stability of Au that endure harsh reaction conditions without compromising the catalytic activities.

## Summary and Outlook

**Figure 6:**
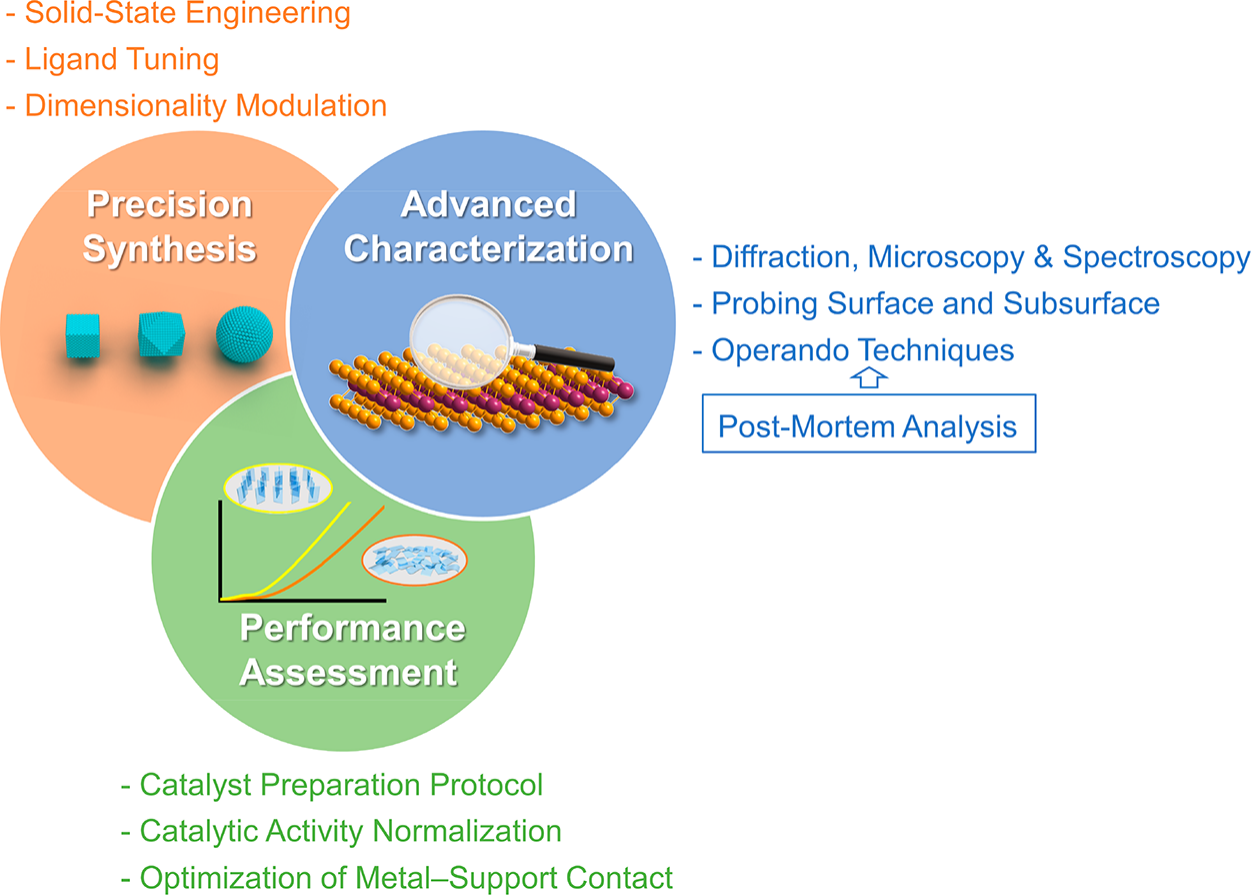
Schematic illustrating future research directions in precision synthesis, advanced characterization, and performance assessment to unveil the structural heterogeneity of nanocatalysts.

The multilevel structural heterogeneities of nanocatalysts afford unprecedented opportunities to obtain mechanistic insights as well as rational optimization of catalytic performances. We herein look into the nanoscale structural heterogeneities involving size and facet tunabilities, differentiation of surface/interface and bulk characteristics, identification of local and average structures, as well as catalytic restructurings under reaction conditions. The subtle difference between the ideal model systems and the realistic complex nanostructures exerts ineligible impact on catalytic behaviors. Future efforts in precision synthesis, advanced characterization and performance assessment are anticipated to tap the full potential for the broad communities of materials and catalysis (Figure 6).

**Figure 7:**
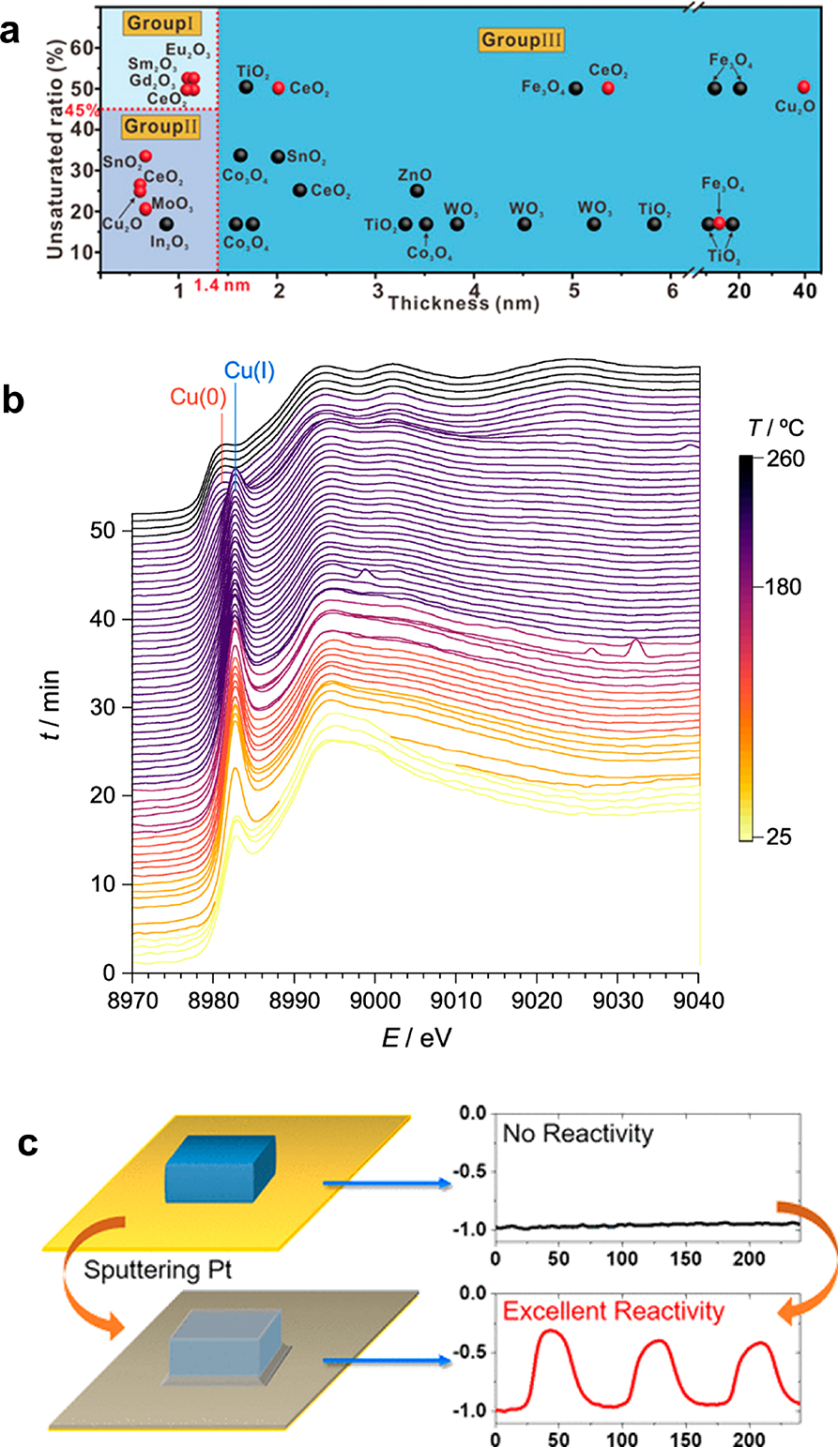
(a) Schematic showing the binary phase diagram of metal oxides, with the surface unsaturated coordination ratio as a function of the thickness of the two-dimensional nanostructures. Reprinted with permission from ref (67). Copyright 2019 John Wiley and Sons. (b) Operando XAS spectra drawn as a function of time, monitoring the evolution of Cu(OAc) to the lamella intermediate and subsequent reduction to metallic Cu. Reprinted from ref (78). Copyright 2022 American Chemical Society. (c) Schematic illustration showing the different activities of the Prussian blue nanoparticles before and after Pt sputtering, highlighting the influence of the interfacial electrical contact. Reprinted from ref (85). Copyright 2020 American Chemical Society.

Precision synthesis of nanostructures, which aims at structure control at the molecular and atomic scale, is the cornerstone for identifying the structural heterogeneities. Colloidal synthesis, which enables delicate tailoring of the nucleation, growth, and stabilization of free-standing nanoparticles in the low-temperature solution phase, emerges as a powerful synthetic toolkit to correlate the atomic structures with catalytic behaviors. Delicate tuning of size, facet, composition, phase, etc. transforms single crystalline surfaces into high-surface-area nanostructures, enabling both mechanistic studies with surface chemistry and performance optimization under realistic reaction conditions.^65^ Moreover, the essence of colloidal synthesis lies in the use of versatile ligand chemistry,^66^ which distinguishes colloidal synthesis from the other catalyst preparation approaches such as impregnation, sol–gel, solvothermal methods, electrodeposition, etc. The long-chain protective ligands promote confined growth of nanocrystals, which may alter the intrinsic dimensionality. For instance, fabricating atomically thin rare-earth metal oxides converts the inherent three-dimensional structure to the quasi-two-dimensional form (Figure 7a).^67^ This dimensionality modulation strategy enables elucidation of surface characteristics, maximization of favorable facets for catalytic conversions, and discovery of new phase structures with appealing catalytic properties. The colloidal nanocrystals can also function as building blocks to access well-designed mesoporous materials. The mass transport and charge transfer in the assembled nanoconfined systems bring extra opportunities for tailoring the catalytic activity and selectivity.^68^

Uncovering the structural heterogeneity of nanocatalysts also requires advances in characterization. Each characterization technique has its own advantages and limitations, which necessitates the combination of a suite of microscopic, diffraction, and spectroscopic tools to unveil the above-mentioned structural details at various scales. For the influence of the size and facet, high-resolution TEM and ADF-STEM enable imaging of the constituent atomic configurations. Building well-defined nanocrystals also allows a quantitative assessment of the morphology and exposed facets using XRD, as demonstrated in the case of shape-controlled TiO_2_ nanocrystals for enhanced photocatalysis.^69^ Synchrotron and neutron diffraction equipped with PDF analyses can bring the discussion to atomically resolved structures. Coupled with microscopic imaging, STEM-EDS and STEM-EELS element maps both permit direct evaluation of the distribution homogeneity of different chemical elements, which is important to identify the local and average structures. EDS is based on the characteristic X-ray generated by the sample upon electron excitation,^70^ while EELS relies on the inelastic scattering interaction between the incident electron and the sample.^71^ Accordingly, STEM-EDS can be more sensitive to metal elements with higher atomic numbers, while STEM-EELS possess higher sensitivities for light elements such as C, N, O, etc.^72^ EELS also enables identification of the oxidation state of the target element, permitting understanding of different redox species from surface to bulk, from center to edge, and across grain boundaries. Note that microscopic images are two-dimensional in nature whereas the real structures are three-dimensional objects. Therefore, three-dimensional reconstruction is needed to accurately identify the position of each individual atom.^73^ For distinguishing surface and bulk structures, X-ray photoelectron spectroscopy (XPS) has been widely applied. However, its capability is weakened for nanoparticles with sizes smaller than that of the probing depth of XPS (∼3–10 nm).^74,75^ In comparison, low energy ion scattering (LEIS) is a more surface-sensitive technique, allowing atomically resolved layer-by-layer analyses for powder and thin-film samples.^76^ The downside is that it is insensitive to the oxidation state of the target elements. For XAS, the highly penetrating power of hard X-ray makes it a bulk characterization technique, but there are certain modes for XAS to become a surface-sensitive technique. For example, the electron yield mode of soft XAS collects electrons within the mean free path of ∼10 Å that is near the surface region. Grazing-incident XAS can also be utilized to extract surface information, where the tunable angle between the incident X-ray beam and the sample enables ease in altering the penetration depth.^77^ These XAS-based developments enable the differentiation of surface and bulk characteristics in nanocatalysts. In the end, in situ and operando techniques such as near ambient pressure XPS (NAP-XPS), diffuse reflectance infrared Fourier transform spectroscopy (DRIFTS), environmental transmission electron microscopy (ETEM), etc. are the panacea to clarify the catalyst restructuring process under reaction conditions. Using in situ XAS and XRD, Buonsanti and co-workers revealed insights into nucleation, growth, and shape control of Cu nanocrystals, showing that the layered coordination polymers as reaction intermediates lead to different shapes of the nanocrystals (Figure 7b).^78^ Such synthetic insights contribute to the fine-tuning and exquisite design of nanocatalysts with size and facet controllability. Note that in most cases, post-mortem analyses of the used or recycled nanocatalysts, which are more operationally feasible, can provide preliminary information to estimate the stability of the reactive surface or its tendency toward restructuring. Surface reconstruction and amorphization, as well as phase transition of the nanocatalysts before and after OER can be systematically examined using TEM (STEM), XRD, and XPS.^52,79,80^

Performance assessment, which is based on the comparison of catalytic performances of a set of nanocatalysts and that of the reference sample, is also important. Catalyst preparation includes the materials synthesis part together with the process transforming the as-synthesized materials to measurable catalysts. In the case of electrocatalysis, catalyst loading or preparation manner leads to different activities even for the same catalysts. For example, as a benchmark HER catalyst that has been well investigated, the Pt/C sample exhibits distinct overpotential values at the 10 mA cm^–2^ geometric area in 0.5 M H_2_SO_4_ solutions.^81,82^ More uncertainties in activity measurement can be expected for the new yet underexplored samples. This brings difficulties in fair evaluation and wise optimization of nanocatalysts, as well as solid demonstration of the validity of design strategies and proposed mechanisms. Report of TOF or activity normalization to the electrochemical surface area (ECSA) is important for gauging and comparing the intrinsic activity of nanocatalysts. Note that the ECSA value, estimated either based on non-Faradaic *C*_dl_ measurement or HUPD, is strongly dependent on the chemical nature and morphology (porosity) of the electrocatalyst as well as the underlying support.^83^ The estimated ECSA value also reflects the number of active sites that are electrochemically active yet not necessarily catalytically active.^84^ Therefore, caution should be taken when turning to ECSA to normalize the electrocatalytic activity. In addition, the error introduced during the preparation of the electrodes are sometimes overlooked. For instance, the electrical contact between the individual supported nanoparticle and the underlying electrode varies, and a poor electrical contact can lead to complete inactivity for the same nanoparticle that would exhibit completely distinct activities with a good contact (Figure 7c).^85^

With these efforts, we may surpass the conventional catalyst systems and embrace the opportunities brought by the increased structural and compositional complexities. For example, chiral nanomaterials serving as a versatile platform to interrogate the transfer and amplification of chirality from molecules to inorganic solids. It has thus aroused extensive interest to realize asymmetric catalysis with desired enantioselectivity on atomically chiral surfaces, whereas controlled creation of chiral atomic configurations for materials that are inherently achiral remains challenging.^86^ High-entropy materials, as another type of emerging materials, are catalytically attractive due to the enhanced configuration entropy confined in the single lattice.^87^ However, questions regarding the influence of long- and short-range order, symmetry breaking at surfaces and interfaces, as well as crystallinity degree in catalytic behaviors limit further mechanistic understanding. Probing and understanding the structural heterogeneity of nanomaterials lay a basic foundation to dive into these sophisticated yet advanced material systems and achieve enhanced catalysis.
